# Nanosomes carrying doxorubicin exhibit potent anticancer activity against human lung cancer cells

**DOI:** 10.1038/srep38541

**Published:** 2016-12-12

**Authors:** Akhil Srivastava, Narsireddy Amreddy, Anish Babu, Janani Panneerselvam, Meghna Mehta, Ranganayaki Muralidharan, Allshine Chen, Yan Daniel Zhao, Mohammad Razaq, Natascha Riedinger, Hogyoung Kim, Shaorong Liu, Si Wu, Asim B. Abdel-Mageed, Anupama Munshi, Rajagopal Ramesh

**Affiliations:** 1 Department of Pathology, University of Oklahoma Health Sciences Center, Oklahoma City, OK, USA; 2 Stephenson Cancer Center, University of Oklahoma Health Sciences Center, Oklahoma City, OK, USA; 3 Department of Radiation Oncology, University of Oklahoma Health Sciences Center, Oklahoma City, OK, USA; 4 Department of Epidemiology and Statistics, University of Oklahoma Health Sciences Center, Oklahoma City, OK, USA; 5 Department of Medicine, University of Oklahoma Health Sciences Center, Oklahoma City, OK, USA; 6 Boone Pickens School of Geology, Oklahoma State University, Stillwater, OK, USA; 7 Department of Urology, Tulane University School of Medicine, New Orleans, LA, USA; 8 Department of Chemistry and Biology, University of Oklahoma, Norman, OK, USA; 9 Graduate Program in Biomedical Sciences, University of Oklahoma Health Sciences Center, Oklahoma City, OK, USA

## Abstract

Successful chemotherapeutic intervention for management of lung cancer requires an efficient drug delivery system. Gold nanoparticles (GNPs) can incorporate various therapeutics; however, GNPs have limitations as drug carriers. Nano-sized cellular vesicles like exosomes (Exo) can ferry GNP-therapeutic complexes without causing any particle aggregation or immune response. In the present study, we describe the development and testing of a novel Exo-GNP-based therapeutic delivery system -‘nanosomes’- for lung cancer therapy. This system consists of GNPs conjugated to anticancer drug doxorubicin (Dox) by a pH-cleavable bond that is physically loaded onto the exosomes (Exo-GNP-Dox). The therapeutic efficacy of Dox in nanosomes was assessed in H1299 and A549 non-small cell lung cancer cells, normal MRC9 lung fibroblasts, and Dox-sensitive human coronary artery smooth muscle cells (HCASM). The enhanced rate of drug release under acidic conditions, successful uptake of the nanosomes by the recipient cells and the cell viability assays demonstrated that nanosomes exhibit preferential cytotoxicity towards cancer cells and have minimal activity on non-cancerous cells. Finally, the underlying mechanism of cytotoxicity involved ROS-mediated DNA damage. Results from this study mark the establishment of an amenable drug delivery vehicle and highlight the advantages of a natural drug carrier that demonstrates reduced cellular toxicity and efficient delivery of therapeutics to cancer cells.

Extensive research in the area of cancer therapeutics has resulted in the discovery and synthesis of many potent small molecule inhibitors with excellent anti-cancer activity^1^,^2^. Despite such tremendous progress, many of these therapeutic molecules have remained at the investigational level, and could not be used for clinical interventions^3^. Conventional therapeutic molecules, such as synthetic drugs, compounds extracted from natural resources, or biomolecules like inhibitory RNA/DNA, do not bear any targeting signals specific to proliferating tumor cells, and produce off-target cytotoxicity^4^. In addition, many of molecules of therapeutic importance are hydrophobic and/or negatively charged, which results in their poor bioavailability to cancer cells^5^,^6^. To circumvent these drawbacks, recent advances in nanotechnology have resulted in the development of various drug delivery vehicles, such as liposomes, polymer-based and inorganic nanoparticles that can be conjugated to signaling molecules and used for targeted tumor therapy^7^,^8^,^9^,^10^. Current delivery systems for anticancer therapeutics are plagued by numerous disadvantages related to low efficiency, poor bio-distribution, and immune response, limiting their application in clinical settings^11^.

Exosomes are submicron-sized cellular vesicles released by cells and can be isolated from all bodily fluids and from the medium of growing cells^12^. Recently, it has been recognized that exosomes can ferry biomolecules, such as nucleic acids and proteins, to the inter-cellular milieu across different membrane barriers without eliciting any immune response^13^,^14^,^15^,^16^. Since exosomes have a structural and functional resemblance to synthetic drug carriers like liposomes, exosomes have recently been investigated for use in drug delivery^17^,^18^,^19^,^20^,^21^. However, poor drug loading and lack of a controlled drug release mechanism are some of the drawbacks of exosome-based drug carriers. Incorporating nanoparticle-drug conjugates with stimuli-responsive properties may overcome the limitations of exosome-based delivery vehicles. Then again, exosomes may provide a non-immunogenic layer protecting the nanoparticle-drug conjugates from rapid clearance and act as a barrier for premature drug release. To prepare nanoparticle-drug conjugates for loading in exosomes, gold nanoparticles (GNPs) may be suitable since they are one of the most studied nanoparticle systems for therapeutic delivery and other biological applications^22^,^23^,^24^,^25^. The smaller size, easy to synthesize, biologically inert and the presence of abundant functional groups for drug conjugation are some the advantages of GNP^26^,^27^,^28^.

The main objective of the present study is to develop an exosome-based drug delivery system for lung cancer treatment. To achieve the objective, we exploited the unique properties and advantages offered by exosomes and GNPs and created a novel exosome-based drug delivery vehicle system called nanosomes. Nanosomes are synthesized by complexing exosomes with NanoDox, which are essentially GNPs conjugated to the anticancer drug doxorubicin (Dox) *via* a pH-sensitive hydrazone linker. The nanosomes were assessed for its therapeutic efficacy against human lung cancer cells, and evaluated the cytotoxic effect in normal cells, especially in doxorubicin-sensitive cardiomyocytes.

## Materials and Methods

### Cell lines and culture conditions

Two non- small cell lung cancer cell lines (H1299, A549) and two normal cell lines namely lung fibroblast cells (MRC9) and human coronary artery smooth muscle cell (HCASM) were used in this study. H1299 and A549 cells were maintained in conditioned (exosome free) RPMI 1640, (GIBCO BRL Life Technologies, NY) supplemented with 10% exosome depleted FBS (System Biosciences, Palo Alto, CA) and 1% penicillin/streptomycin. While MRC9 cells were cultured in conditioned (exosome free) EMEM media (GIBCO BRL Life Technologies, NY) supplemented with 10% exosome free FBS (System Biosciences, Palo Alto, CA) and penicillin/streptomycin as described above. For culturing HCASM cells, vascular cell basal medium supplemented with vascular smooth muscle cell growth kit was used as per recommendation of ATCC except for FBS which was replaced with 10% exosome free medium. All cells were purchased from American Type Culture Collection (ATCC, Manassas, VA).

### Purification of exosomes

To isolate exosomes, H1299 and YRC9 cells were cultured in their respective conditioned medium, as described above, until 80–90% confluence was achieved. The conditioned medium was then collected for isolation of exosomes using a modified method of Thery *et al*.^29^ as described in supplementary section 1.

### Synthesis of Nanosomes

Gold nanoparticles (GNPs) were synthesized using the sodium citrate-mediated reduction method^30^. Briefly, 20 ml of 1 mM gold chloride (HAuCl4; Sigma Aldrich Chemicals St. Louis, MO) was stirred on a hot plate (80 °C) at high speed. Three ml of 1% trisodium citrate was added and stirring continued until the solution began to show a color change from yellow to wine red, which indicates the formation of gold nanoparticles. Conjugation of doxorubicin (Dox; Sigma Aldrich) to GNP via a pH sensitive linker was achieved as previously described^22^ and the Dox conjugated to GNPs was termed as Nano-Dox.

To make nanosomes, NanoDox (with 35 μg Dox equivalent) was co-incubated with an average of 3E + 14 particles (equivalent to 400 μg of protein as measured by BCA assay) of exosomes in an incubator at 37 °C with 250 rpm for 2 h. The nanosomes were then purified using the ExoQuick-TC^®^ kit (System Biosciences) followed by centrifugation at (1500× g for 30 min. The nanosome pellet thus obtained was re-suspended in PBS (pH 7.4) and stored in −20 °C until used.

### Characterization of exosomes

The exosomes isolated from cell cultures were characterized for their size, shape concentration and for presence of membrane proteins. The details of the methods used for exosome characterization is described in supplementary section 2.

### Characterization of Nanosomes

#### Zeta potential, size, and structure

The zeta potential of each step of nanosome synthesis was measured using the Zeta PALS (Brookhaven Instruments, Holtsville, New York). The size and structure of GNPs and nanosomes were analyzed by using transmission electron microscopy (TEM).

#### Estimation of Dox in NanoDox and Nanosomes

After conjugation of Dox to the GNP through the pH linker, the unbound Dox was collected after washing with PBS. The unbound Dox in the supernatant was measured at 485 nm absorbance using DeNovix^®^ DS-11 spectrophotometer (Denovix Inc. Wilmington, DE). The values obtained from the supernatant were used to estimate the amount of Dox present in the NanoDox by comparing with known Dox concentrations. Similarly, after nanosomes synthesis, the unbound NanoDox was collected and incubated overnight with 1 M HCl in a 2:1 ratio to cleave free Dox from NanoDox. After the incubation was complete, the solution was centrifuged at 100,000× g for 1 h and the supernatant was carefully collected. The amount of Dox present in the supernatant was measured at 485 nm absorbance and estimated the bound Dox in the nanosomes by comparing this with known Dox concentrations.

#### Estimation of gold in NanoDox and nanosomes

Inductive Coupled Plasma Mass Spectrometer, (ICP-MS; iCAP^TM^ Q, Thermo Scientific, Waltham, MA) was used to estimate the elemental gold (Au) content in the NanoDox and nanosome preparation. Two hundred μl of freshly prepared NanoDox particles and nansosomes were digested in 5% Aqua regia (1:3 v/v, of HNO_3_: HCl) overnight. The next day, after making appropriate dilutions with double deionized water, sample aliquots were analyzed for Au element concentration via ICP-MS. The percent of Au present in the nanosomes was calculated using control spiked with a known Au concentration.

Further, ICP-MS was also used to determine the Au concentration in the H1299 cells treated with nanosomes. Briefly, after 24 h of treatment, the cells were harvested and washed three times with PBS (pH 7.4) to remove physically bounded particles and subsequently digested in 5% Aqua regia and assayed as described above.

### *In vitro* release studies of Dox from NanoDox and nanosomes

To estimate the release of Dox from the NanoDox and nanosomes in response to pH stimuli, the complexes were suspended in phosphate buffer (PBS; pH 7.4) and acetate buffer (ABS; pH 5.5), to imitate the physiological and tumor microenvironments, respectively. Briefly, 200 μl of NanoDox and nanosomes suspended in PBS and ABS buffers were incubated for predetermined time points at 37 °C with shaking at 220 rpm. At each time point specified, the suspensions were centrifuged (22,000× g, 45 min) and the supernatants were collected in a fresh tube for fluorescence measurement at 485 nm excitation and 535 nm emission wavelength using EnVision^®^ multilabel reader (Perkin Elmer Life and Analytical Sciences, Shelton, CT). The residual pellet was again re-suspended by adding equal volume of respective buffers and the same procedure was continued for each time point of study. The amount of Dox released from NanoDox and nanosome was calculated from the known starting concentration of Dox in respective formulations and the values are represented as percentage of Dox released over time.

### Cellular uptake studies

H1299 lung cancer cells were seeded in a six-well plate at a density of 1 × 10^5^ cells/well. Cells were treated with free-Dox, NanoDox, and nanosomes containing the equivalent of 5 μg Dox/well, while an untreated group was kept as controls. After 24 h of treatment, uptake of the complex by recipient cells was measured at three levels representing the three components of nanosomes *viz* exosomes, GNP and Dox.

#### Fluorescence microscopy

5 × 10^4^ cells were seeded on cover-slips in a six-well plate and were treated with NanoDox, nanosomes and free-Dox. One additional group included in the study was GFP-labeled exosomes produced by H1299 cells that was stably transfected with GFP-CD63 plasmid and loaded with NanoDox (Nanosome-GFP) (supplementary methods). After 24 h of treatment, the cells were washed in ice-cold PBS, followed by fixation with 4% paraformaldehyde (PFA). DAPI (4′,6-diamidino-2-phenylindole) was added to stain nuclei. Images were acquired using a Nikon epifluorescence microscope (Nikon Instruments, NY).

#### Estimation of GNP

The estimation of Au content in H1299 cells treated with nanosomes was done by ICP-MS as described previously in the section 2.5.3.

#### Fluorescence measurement of Dox

After 24 h of treatment with NanoDox, nanosomes, free-Dox, and exosomes, cells were harvested and washed with PBS in a 15 ml conical tube. The collected cells were sonicated and subjected to Dox fluorescence measurement at 535 nm emission wavelength using an EnVision^®^ multi-plate reader system (PerkinElmer). Fluorescence intensity normalized to 10,000 cells for each group was calculated from the readings obtained from the plate reader. Further, a full fluorescence spectrum from 500 nm to 700 nm was measured using SpectraMax M2 instrument (Molecular Devices, Sunnyvale, CA) to obtain the peak for Dox in the treated cell lysates.

### Cell viability assay

For cell viability experiments 1 × 10^5^ cells per well were seeded in six-well plates. After 24 h of incubation, the culture medium was replaced with serum free medium and the cells were subjected to appropriate treatments. Six-hours after treatment, the culture medium was replaced with complete medium supplemented with 5% FBS. At 24 h after treatment, the cells were collected and viability determined by the Trypan blue exclusion assay method^23^. In all of the studies, cells receiving no treatment served as controls.

Prior to determining the therapeutic efficacy of the nanosomes, we first determined the exosome source and concentration for preparing nanosomes. H1299 cells were treated with three different concentrations (24, 60 and 96 μg/well) of exosomes derived from H1299 and MRC9 cells and the effect of exosome treatment on cell proliferation was evaluated at 24 h after treatment.

To determine the optimal dosage of Dox in nanosomes needed for therapeutic studies, H1299 cells were treated with NanoDox and nanosomes at six different Dox concentrations (1, 2, 3, 4, 5, 8, and 10 μg Dox/well) and cell viability was assessed at 24 h after treatment.

Finally, the therapeutic effect of nanosomes, Nano-Dox and free Dox on lung cancer (H1299 and A549) and normal (MRC9 and HCASM) cell lines were determined. Equivalent dosage of 5 μg/well Dox was present in each of the treatment group. Treated cells were harvested at 24 h after treatment and the number of viable cells determined. Further, to investigate the long term effect of nanosomes treatment, cells viability was determined at different time points (24, 48 and 72 h) and compared to NanoDox in H1299 cells.

### Cell cycle analysis

Lung cancer (H1299, A549) and normal (MRC9) cells (1 × 10^5^ cells/well) seeded in individual six-well plates were treated with NanoDox, nanosomes and free Dox (5 μg of equivalent Dox) in each well. Untreated cells served as control group. Twenty-four hours after treatment, the cells were collected, washed with ice cold PBS and stained with propidium iodide (Sigma Aldrich) and subjected to flow-cytometric analysis (FACS Calibur; Becton Dickinson, San Jose, CA) as previously described^31^.

### Western blotting

Total cells lysates prepared from lung tumor and normal cells receiving various treatments were subjected to Western blotting as previously described^32^ and is briefly illustrated in supplemetry section 3.

### Comet assay

Double strandbreaks (DSBs) induced by free-Dox, NanoDox and nanosomes were determined by Comet assay (CometAssay^®^ kit; Trevigen, Inc., Gaithersburg, MD) as previously described^32^. The detail of the comet assay is outlined in supplementary section 4.

### Mitochondrial perturbation assay

H1299 and MRC9 cells (5 × 10^4^ cells/well) seeded in chamber slides were treated with, NanoDox, nanosomes, and free-Dox containing 5 μg of equivalent Dox/well. After 24 h of treatment, the cells were stained using cationic Dye JC-1 (Sigma Aldrich) as recommended by the manufacturer’s protocol. Briefly, the cells were incubated with JC-1 staining solution for 20 min at 37 °C. After completion of the incubation, the staining solution was aspirated and the cells were washed twice with culture medium. The cells were subsequently overlaid with fresh culture medium and observed under an inverted Leica SP2 MP confocal microscope (Leica Microsystems, Buffalo Grove, IL). JC-1 aggregate and JC-1 monomer fluorescence was determined by excitation/emission at 525 nm/590 nm and 490 nm/530 nm wavelength respectively.

### Reactive oxygen species (ROS) assay

H1299 and MRC9 cells (1 × 10^5^) were treated with NanoDox, nanosomes and free-Dox containing 5 μg of equivalent Dox/well. After of 24 h treatment the cells were washed with 1X Hanks balanced salt solution (HBSS), and incubated with 20 μM 2′,7′-dichlorofluorescein diacetate (DCFDA) dye (Molecular Probes, NY) in fresh HBSS for 60 min at 37 °C. Untreated cells incubated with DCFDA were used as controls to subtract the background DCFDA fluorescence. The cells from each treatment group were carefully collected by scrapping and the ROS levels were determined by measuring the fluorescence using the EnVision^®^ multi-plate reader system (PerkinElmer) at 485 nm excitation and 535 nm emission wavelength. The fluorescence intensity (F.I.) representing ROS levels per 10,000 cells were plotted after normalizing the fluorescence from control cells. The results obtained are shown as the average F.I. ± SD and were subjected to statistical analysis.

For determining the contribution of nanosome and NanoDox treatment in inducing ROS, a ROS quenching assay was performed. Briefly, cells (H1299 and MRC9) seeded in six-well plates were treated with NanoDox and nanosomes as described above for ROS assay. At 24 h after treatment the cells were incubated with 20 μM DCFDA alone or incubated with a combination of DCFDA and the antioxidant, *N*-Acetylcysteine (2 mM; NAC; Sigma Aldrich) for 60 min at 37 °C. All other experimental condition and analysis performed was identical to that described for ROS assay.

### Statistical analysis

All the experiments were carried out in triplicates and all data are shown as mean ± standard deviation (*SD*). Outcome variables including exosomes, cell viability and fluorescence intensity were compared among treatment groups using one-way ANOVA. The p-values for pairwise comparisons were adjusted using the Tukey’s method. Adjusted p-values less than 0.05 were considered to be statistically significant. SAS 9.4 was used for performing statistical analysis.

## Results

### Exosome isolation and characterization

Exosomes were purified from conditioned media obtained from culturing H1299 and MRC9 cells by using the differential high speed and ultracentrifugation method. To ascertain the presence of exosomes in the isolated preparations, we followed the recommendations of the International Society of Extra Cellular Vesicles (ISEV)^33^. We first measured the concentration and size of exosomes with an Izon particle analyzer system. This analysis revealed that the isolated vesicles were 50–200 nm in size, with the majority of the vesicle population in the diameter size range of 70–110 nm. The mean size of vesicles was 86 nm (Supplementary Fig. 1A). The average concentration of exosomes was 5.0 × 10^11^ particles per ml. Next, the size and structure of purified exosomes was confirmed by TEM imaging and showed typical bilayer membrane, vesicles of size less than 100 nm (Supplementary Fig. 1B). Lastly, exosome isolation was confirmed at the molecular level by probing the isolates with exosome-associated protein markers. Western blot analysis revealed that membrane proteins, tetraspanins CD63 and CD81, and TSG101, a member of the ESCRT-1 complex of the vesicular transport system, were elevated in exosome samples compared with respective cell lysates. In contrast the Hsp90B1(Grp94) and AGO2 cytosolic proteins were not detected in the exosome samples but were present in H1299 and MRC9 cell lysates (Supplementary Fig. 1C). Hsp90B1(Grp94) and AGO2 are not expected to be enriched in the exosomes per ISEV guidelines. The data obtained from all three analyses fulfilled the three criteria set by ISEV, and established the presence of exosomes in the isolates.

### Synthesis and physicochemical characterization of nanosomes

**Figure 1:**
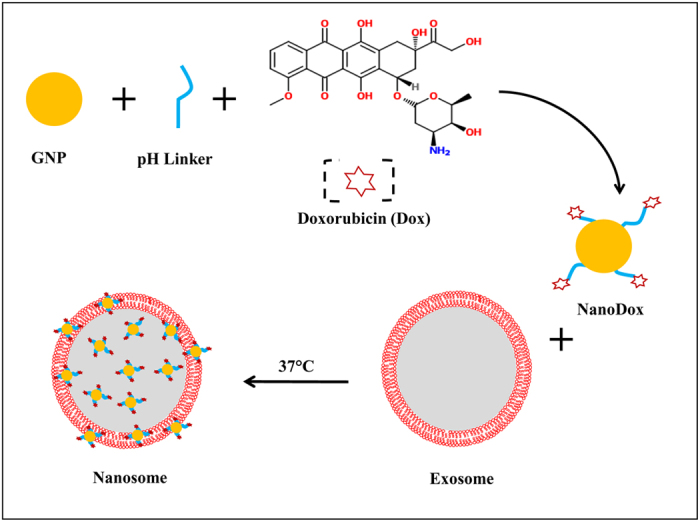
Schematic of nanosome synthesis.

The schematic shows the major steps involved in the synthesis of nanosomes (Fig. 1).

**Figure 2:**
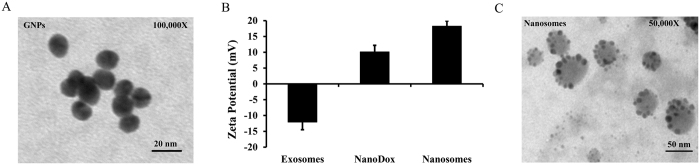
Characterization of nanosomes. (**A**) TEM image of free gold nanoparticles (GNPs); Scale bar, 20 nm. (**B**) Change in average Zeta potential of exosomes as a result of its complexation with NanoDox to form nanosomes. (**C**) TEM images of nanosomes: the dark black spherical spots indicate GNP-Dox particles; scale bar, 50 nm.

First, GNPs were synthesized by a chemical reduction method using trisodium citrate. TEM imaging showed that the GNPs had a well-dispersed, high-density, spherical structure and had an average size of 10 nm (Fig. 2A). Next, HS-PEG-OMe was attached to the GNPs through Au-S linkage to avoid agglomeration and was finally conjugated with Dox through a pH sensitive hydrazone linker to form NanoDox (GNP-Dox). Absorbance spectral analysis of NanoDox at 485 nm confirmed the presence of Dox and determining the loading efficiency of Dox revealed 71%. Following the confirmation of successful NanoDox synthesis, they were loaded into MRC-9-derived exosomes by incubating at 37 °C to form nanosomes. The loading of NanoDox into exosome produced a change in the surface charge of the exosome (Fig. 2B and Supplementary Fig. S-2A). The net surface charge of NanoDox was +10.13 ± 0.9 mV due to the presence of cationic Dox, while the exosomes showed a negative surface charge of −12.07 ± 2.4 mV. However, upon loading of NanoDox into exosome the surface charge shifted from a negative to positive charge and became +18.24 ± 1.67 mV. This change in the zeta potential indicated successful transition of exosomes to nanosomes. (Fig. 2B and Supplementary Fig. S-2A).

Further to visually verify the successful preparation of nanosomes and to examine any changes that might have occurred in the structure of exosomes due to loading of NanoDox, TEM imaging was performed. The images obtained show the presence of dense black NanoDox particles on the exosomes (i.e. nanosomes). The images show well-dispersed nanosomes. No change in exosome size or shape was observed before or after loading with the NanoDox complex, suggesting that the loading method does not cause any perturbation in the exosome structure (Fig. 2C and Supplementary Fig. S-2B). Finally, the loading of NanoDox into exosomes was confirmed by estimating the Au content in the nanosomes by ICP-MS. Approximately 236 particle per billion (ppb) of Au was present in the nanosomes (Table 1), which is equivalent to 29% of NanoDox loaded onto the exosomes when compared to the initial Au content used during the synthesis of NanoDox.

### Drug release kinetics from NanoDox and nanosomes

**Figure 3:**
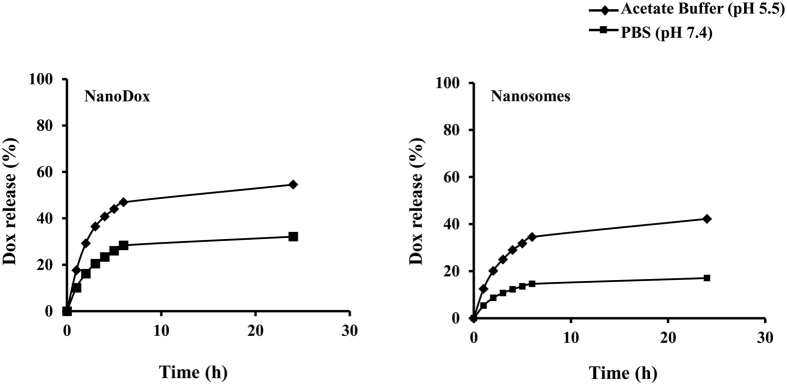
*In vitro* release kinetics of Dox was estimated in two buffers: (1) PBS, with physiological pH (7.4) and acetate buffer (ABS), with acidic pH (5.5) similar to the pH present in the tumor microenvironment.

Dox released from NanoDox and nanosomes was examined in phosphate buffered saline (PBS; pH 7.4) and acetate buffer (ABS; pH 5.5). The Dox release was estimated by measuring the fluorescence of Dox at pre-determined time intervals in ABS and PBS up to 24 h. The results show that the release rate of Dox from both NanoDox and nanosomes was markedly higher under acidic pH conditions (ABS; pH 5.5) than under physiological pH conditions (PBS; pH 7.4). The observed difference is attributed to the acid-labile hydrazone pH linker present in NanoDox. Interestingly, the fluorescence intensity measurement showed a higher percentage of drug release from NanoDox (54.5% in ABS, and 32.13% in PBS) than from nanosomes (42.1% in ABS and17.5% in PBS) at 24 h of incubation (Fig. 3). The lower rate of drug release from the nanosomes can be attributed to the delay in the exposure of NanoDox that are trapped in the exosome to the external buffered environment. In contrast, NanoDox are in direct contact with the buffers as soon they are added into the buffer solution resulting in rapid drug release. This differential and slow drug release from nanosomes is beneficial for cancer therapy as it is likely to retard rapid elimination of the drug from the body when administered *in vivo*.

### Nanosomes are efficiently taken up by the cancer cells

**Figure 4:**
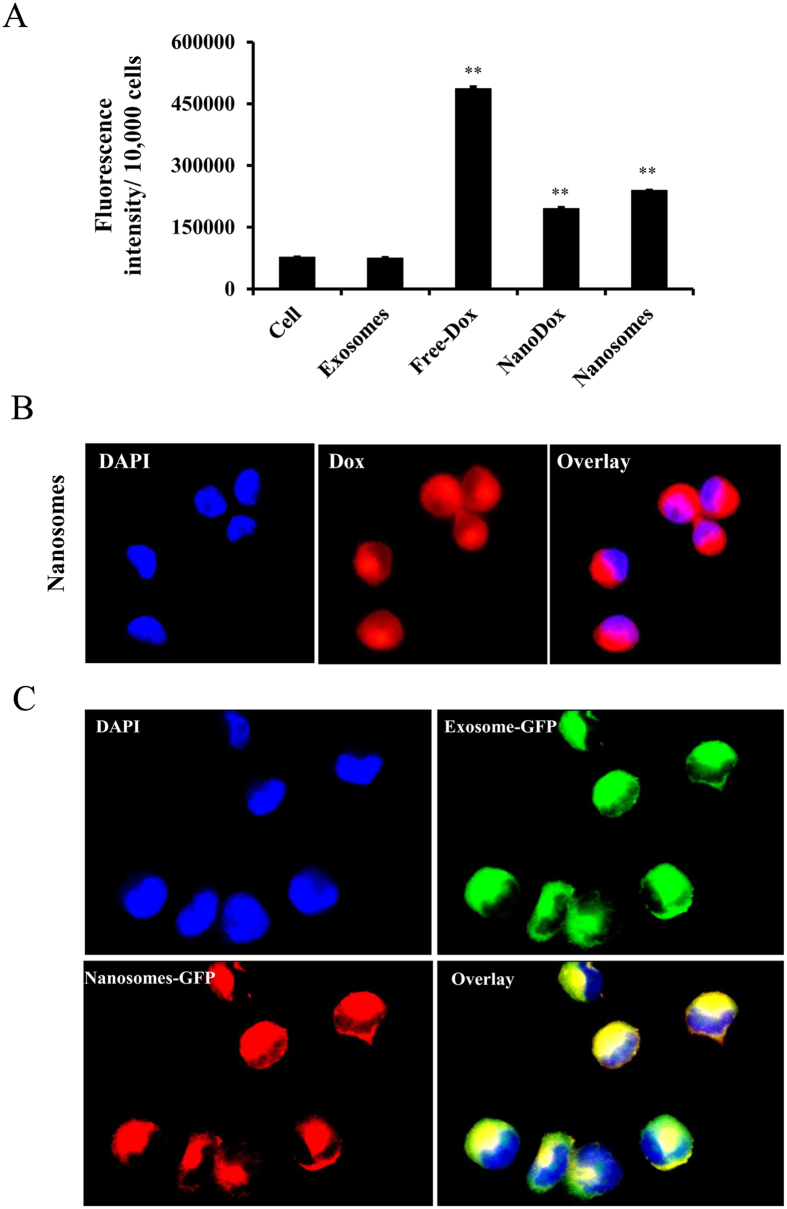
Cellular uptake of nanosomes. (**A**) Fluorescence intensity (FI) measuring Dox uptake after 24 h of treatment of H1299 cells with free-Dox, NanoDox and nanosomes, compared with untreated control cells and exosomes. (**B**) Epifluorescence images of nanosomes-treated H1299 cells showed colocalization of red fluorescence (due to Dox) to the nucleus (DAPI), suggesting the uptake of nanosomes by the recipient H1299 cells. (**C**) Epifluorescence images showing GFP-labeled exosomes (green) loaded with NanoDox (nanosomes; red) are efficiently taken up by H1299 cells (overlay).

After demonstrating successful incorporation of NanoDox into the exosomes to form nanosomes, the next step was to investigate whether the nanosomes could successfully enter the recipient cells and efficiently deliver the therapeutic cargo. To assess the uptake, H1299 cells were treated with nanosomes and NanoDox containing the equivalent of 5 μg Dox per well for 24 h. As shown in Fig. 4A, higher uptake of nanosomes as evidenced by the fluorescence intensity was observed compared to NanoDox, Dox-free exosome, and no treatment control (*p* < 0.05). The highest fluorescence intensity however was observed with free-Dox that served as a positive control. The nanosome uptake by H1299 cells was further substantiated by fluorescence spectrum analysis that demonstrated the expected fluorescence peak for Dox at 590 nm that was relatively larger than in NanoDox-treated cells (Supplementary Fig. S-3). The explanation for the observed enhanced cellular fluorescence intensity in the nanosomes-treated group compared to NanoDox treated group is attributed to higher nanosome uptake by the cancer cells. The cells treated with Dox-free exosomes showed fluorescence similar to that of untreated control cells, which indicates that the exosomes themselves did not contribute to any fluorescence.

Cell uptake of nanosomes was also confirmed by detecting fluorescence emitted by Dox with fluorescence microscopy. As shown in Fig. 4B, the nucleus and cytoplasm in nanosome-treated cells displayed intense red fluorescence which indicates the presence of nanosome inside the cells and successful Dox delivery. However this result can also be interpreted as indicative of Dox uptake independent of nanosome uptake. Hence to negate this premise, a green fluorescent protein (GFP)-labeled exosome (GFP-exosome) loaded with NanoDox (GFP-Nanosome) was also used and determined its uptake by H1299 cells. As shown in Fig. 4C, co-localization of red (Dox) with green (GFP-exosome) fluorescence in the nucleus and cytoplasm of the recipient unlabeled H1299 cells confirms the successful uptake of nanosomes by the recipient H1299 cells.

The cell uptake was further evaluated by estimating the Au content in nanosome-treated cells using ICP-MS. The results show that 89.02 ppb of Au was present in the cells treated with nanosomes which is equivalent to the 37% of the initial amount of Au present in nanosomes (Table 1). In contrast, only 14.55 ppb of Au (1.4%) was detectable in the cells treated with NanoDox. All of the results convincingly demonstrate that nanosomes are efficiently taken up cancer cells.

### Selection of exosome source to synthesize delivery vehicle

The successful physical and physiological characterization of nanosomes led us to assess their functional role as a therapeutic delivery vehicle. Hence, we examined the feasibility of using H1299 cells-derived exosomes to synthesize a nanosome complex intended to be used as therapeutic delivery vehicle and compared to MRC9 cells-derived exosomes. Three different concentrations of exosomes purified from H1299 and MRC9 cells were tested for their effect on H1299 cell proliferation in a cell viability experiment. After 24 h of treatment with MRC9 cells-derived exosomes, no significant changes in cell viability were observed at any of the three concentrations, compared with untreated control cells (Supplementary Fig. S-4). In contrast, treatment with H1299 cells-derived exosomes, yielded progressive and statistically significant increases in cell proliferation at exosome concentrations of 24 (98%, *p* = 0.24), 60 (113.9%, *p* < 0.05), and 96 μg (120.8%, *p* < 0.05) per well, compared with untreated control cells (Supplementary Fig. S-4). This result indicated that normal fibroblast-derived exosomes but not cancer-cell derived exosomes will be beneficial for developing and testing nanosomes for drug delivery purposes. Hence, all of the studies described hereafter utilized MRC9 cells-derived exosomes to synthesize nanosomes and were used in efficacy and functional studies.

### Optimization of Dox concentration in nanosomes

To evaluate the therapeutic efficacy of nanosomes prepared using MRC9-derived exosomes, we first determined the optimum concentration of Dox to be used for viability assays. We evaluated different concentrations of Dox (1 –10 μg) for use with NanoDox and nanosomes in H1299 cells. A dose-dependent cytotoxicity was observed with both NanoDox and nanosomes after 24 h of treatment (Supplementary Fig. S-5A and B). A Dox concentration of 10 μg/well yielded the maximum toxicity for both NanoDox (37.64%; *p* < 0.0001) and nanosome (37.05%; *p* < 0.0001) compared to untreated control. At a concentration of 5 μg/well, approximately 50% of the cells were viable in both NanoDox and nanosome treatment group compared to untreated control. Based on this result, we chose a NanoDox and nanosome concentration containing the equivalent of 5 μg Dox/well for further experiments the results of which are described below.

### Nanosomes exert selective cytotoxicity to lung cancer cells but not normal fibroblast cells

**Figure 5:**
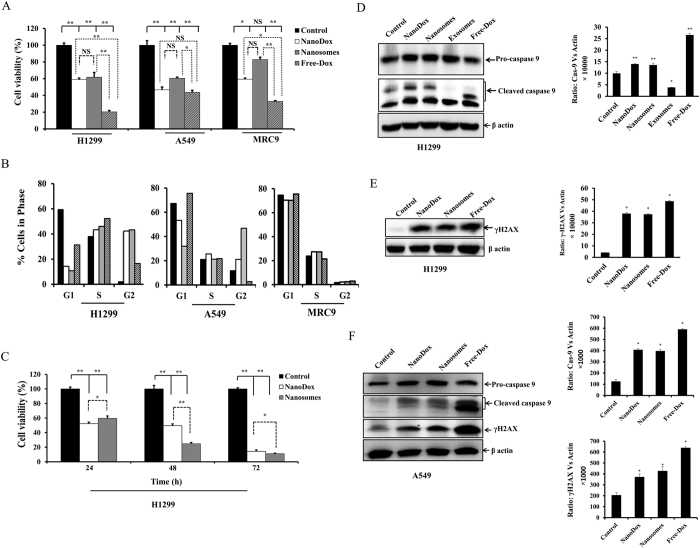
Nanosomes exhibit selective cytotoxicity to lung cancer cells but normal cells. (**A**) Cell viability of two lung cancer (H1299 and A549), and normal (MRC9) lung fibroblast cell lines after 24 h of treatment with NanoDox, nanosome, and free-Dox, each containing the equivalent of 5 μg Dox/well. Each bar represents the percent viable cells after 24 h of treatment ± *SD* compared with untreated control. *Represents *p* < 0.05, **represents *p* < 0.0001, and NS = not significant. (**B**) Cell-cycle analysis shows induction of G2 phase cell-cycle arrest in NanoDox and nanosome-treated H1299 and A549 cells but not MRC9 cells at 24 h after treatment. (**C**) Viability of H1299 cells after treatment with NanoDox and nanosomes, each containing the equivalent of 5 μg Dox/well at 24, 48, and 72 h. Each bar represents the percent viable cells after treatment at respective time points ± *SD* compared with untreated controls. *Represents *p* < 0.05, **represents *p* < 0.0001. (**D**) Western blotting analysis for caspase-9, as an indicator of apoptosis, in H1299 cells after 24 h of treatment shows marked activation of caspase-9 in NanoDox, nanosome and free Dox-treated cells compared to control. (**E**) NanoDox, nanosome and free-Dox-treated H1299 cells show increased γH2AX expression indicating cell undergoing DNA damage compared to control. β-actin was used as a loading control. (**F**) Activation of caspase-9 and increased γH2AX expression in A549 cells treated with NanoDox, nanosomes and free-Dox compared to controls. β-actin was used as a loading control. *Represents *p* < 0.05, **represents *p* < 0.0001.

We performed cell viability tests in two lung cancer (H1299 and A549) and one lung fibroblast (MRC9) cell lines. The viability assay was conducted with NanoDox, nanosomes and free-Dox having the equivalent of 5 μg Dox/well in each treatment. Cells that did not receive any treatment served as controls. After 24 h of treatment, a significant reduction in viability in both H1299 and A549 cells was observed in all of the treatment groups compared to control (Fig. 5A; *p* < 0.05). In H1299 cells, free-Dox produced the maximum inhibitory effect (20.3% viability; *p* < 0.0001) compared to control, NanoDox (58.8% viability; *p* < 0.0001), and nanosomes (61.7% viability; *p* < 0.05). Although, NanoDox exhibited slightly enhanced toxicity compared to nanosomes, there was no significant difference between the two treatment groups (*p* = 0.68). In A549 cells akin to H1299 cells, a significant reduction in cell viability was observed in both NanoDox (46.6% viability; *p* < 0.05) and nanosome-treated (60.5% viability; *p* < 0.05) groups compared to control (Fig. 5A). However, NanoDox exhibited greater inhibitory activity than nanosomes on A549 cells (*p* < 0.05). Although free-Dox showed the highest cell killing efficiency (43.5% viability, *p* < 0.0001), no significant difference was observed between free Dox and NanoDox (*p* = 0.49). A significant difference in cell viability was observed between free Dox and nanosomes, and between NanoDox and nanosomes (*p* < 0.005; Fig. 5A).

In MRC9 cells, a significant reduction in cell viability was observed in both free-Dox (32.8% viability; *p* < 0.001) and NanoDox (59.3% cell viability, *p* < 0.05) treatment groups compared to control (Fig. 5A). In contrast, only 11.2% reduction in cell viability was observed when treated with nanosome (82.8% viability*; p* = 0.16) compared to control. These results demonstrate that nanosomes selectively reduce tumor cell viability and spare normal cells. Hence, nanosomes may be more effective in cancer treatment by minimizing the non-specific toxicity compared to free-Dox and NanoDox treatments.

Next we determined the treatment effects of nanosomes on cell cycle and compared with all other treatment groups. As shown in Fig. 5B, an increase in the number of cells in the G2 phase of cell cycle was observed in both nanoDox and nanosome-treated H1299 and A549 cells when compared to untreated controls. Treatment with free-Dox however showed increased G2 arrest in H1299 cells and increased G1 arrest in A549 cells when compared to untreated control cells (Fig. 5B). The underlying difference for G1 versus G2 arrest induced by free Dox between the two cell lines is not clear and is to be determined. In MRC9 cells, no marked change in G2 phase was observed in nanoDox and nanosome-treated MRC9 cells when compared to untreated controls (Fig. 5B). Also, no difference in G1 or G2 was observed when treated with free-Dox and compared to untreated control.

Since, nanosome-treatment exhibited increased cytotoxicity to tumor cells at 24 h after treatment, we investigated the cytotoxicity of nanosomes over time and compared to NanoDox in H1299 cells. Cell viability studies were conducted at 24, 48, and 72 h with NanoDox and nanosomes containing the equivalent of 5 μg of Dox per well and compared with untreated control. Both, NanoDox and nanosome significantly reduced cell viability at all three time points tested compared to control (Fig. 5C; *p* < 0.05). Comparison between NanoDox and nanosome showed NanoDox initially showed a greater inhibitory activity on cell viability (52.3%) compared to nanosome (59.7%) at 24 h. However, nanosomes exhibited greater inhibitory activity at 48 h (24.8% viability; *p* < 0.0001;) and 72 h (11.1% viability; *p* < 0.05) compared to NanoDox (49.4% viability at 48 h and 14.3% viability at 72 h). This result shows that nanosomes permit the slow and sustainable release of Dox at a slightly higher rate than that of NanoDox implying a controlled and not burst release of drug in the cell after treatment, a feature that is preferred in cancer treatment. Finally, our observations strongly favor nanosomes as a better drug delivery carrier than NanoDox.

The possibility that free-Dox-loaded exosomes (Exo-Dox) might be a better carrier than nanosomes was also investigated. As shown in Supplementary Figure S-6A, drug-release kinetics from Exo-Dox exhibited a burst release of Dox in both physiological (pH 7.4) and acidic buffer (pH 5.5) conditions that were in stark contrast to the slow and sustained release kinetics observed for nanosome (Fig. 3). The burst drug release correlated with the heightened reduction in cell viability in Exo-Dox-treated H1299 cells compared to nanosome-treated cells (Supplementary Figure S-6B; *p* < 0.0001). While the enhanced killing observed with Exo-Dox might be considered favorable, it is to be realized that for effective cancer cell killing a slow and sustained drug release is preferred thus making nanosome a preferred drug delivery carrier.

### Nanosome treatment activates caspase-9 and induces DNA damage in lung cancer cells

Doxorubicin is a well-known apoptosis inducer and intercalates into DNA causing DNA damage and disruption in DNA repair^34^. Cysteine-dependent aspartate-specific proteases-9 (caspase-9) is one of three caspases involved in apoptosis signal transduction. The extent of apoptosis is indicated by cleaved caspase-9, which denotes activation of apoptosis signaling through mitochondrial pathway^35^,^36^. Figure 5D shows that NanoDox, nanosomes and free-Dox-treatment resulted in the activation of caspase-9 in H1299 cells. Cleaved caspase-9 was significantly higher in NanoDox, nanosomes and free Dox-treatment groups when compared to control and exosome treatment groups (*p* < 0.001). The highest caspase-9 cleavage however was observed in free-Dox-treatment group. Caspase-9 cleavage was negligible in exosome treatment alone when compared to control. These results indicate that the Dox carried by nanosomes was able to efficiently induce apoptosis.

Since phosphorylation of H2AX (γH2AX) indicates early signs of DNA damage^37^, we analyzed the ability of nanosomes in inducing γH2AX phosphorylation. A marked increase in phosphorylated γH2AX expression was observed in H1299 cells treated with nanosomes, Nanodox and free-Dox compared to the untreated control (Fig. 5E; *p* < 0.05). No significant differences in γH2AX expression between NanoDox and nanosome treatment groups were observed. The highest γH2AX however was observed in free-Dox-treated cells that concurred with the capase-9 cleavage data indicating free-Dox exerted the maximum DNA damage.

A similar trend in caspase-9 cleaved products and γH2AX expression was also observed in A549 cells when treated with NanoDox, nanosomes, and free-Dox (Fig. 5F; *p* < 0.05). The highest capase-9 and γH2AX activity was observed in free-Dox treatment akin to that observed in H1299 cells.

**Figure 6:**
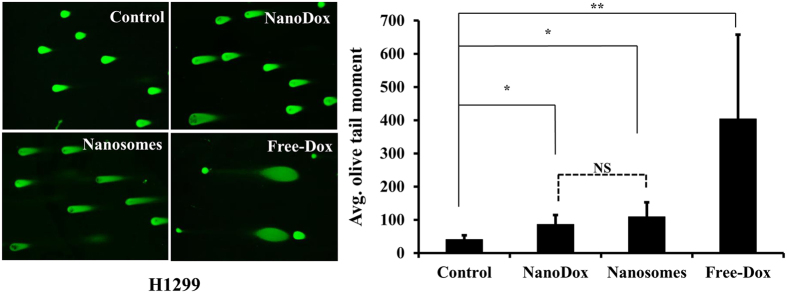
Nanosome-treatment produces DNA damage. (**A**) Photomicrographs showing DNA damage as visualized by the extent of the tail length of the comet in NanoDox-, nanosome-, and free-Dox-treated cells. Cells receiving no treatment served as controls. (**B**) The quantitative representation of the Olive tail moment showing significant DNA damage produced by NanoDox and nanosome treatments compared to control. Greatest DNA damage was produced by free-Dox. *Represents *p* < 0.05, **represents *p* < 0.0001, NS = not significant.

Although γH2AX measurement indicates occurrence of DNA damage, it does not provide information on the extent of damage. Therefore, to determine the extent of DNA damage we performed neutral comet assay in H1299 cells. In this assay, the measure of fluorescence intensity of the head-to-tail length of a comet-like structure formed by damaged nuclei (olive tail moment) represents the extent of DNA damage^38^, and hence measures the cytotoxic effects of Dox. As shown in Fig. 6, free -Dox-treated H1299 cells showed the greatest DNA damage, producing the largest head-to-tail length and maximum olive tail moment compared with nanosomes, NanoDox, and untreated cells (*p* < 0.0001). Cells treated with nanosomes and NanoDox also showed significant DNA damage compared to control (*p* < 0.05). The extent of DNA damage although not statistically significant was observed to be higher in nanosome-treated cells than in NanoDox-treated cells. The increased DNA damage induced by nanosomes compared with NanoDox can be explained as an outcome of increased cell uptake, as shown in Fig. 4. Our results indicate that nanosomes like free-Dox and NanoDox can efficiently activate apoptosis and induce DNA damage in lung cancer cells.

### Nanosomes induce reactive oxygen species (ROS) and perturb the mitochondria in lung cancer cells but not normal fibroblast cells

**Figure 7:**
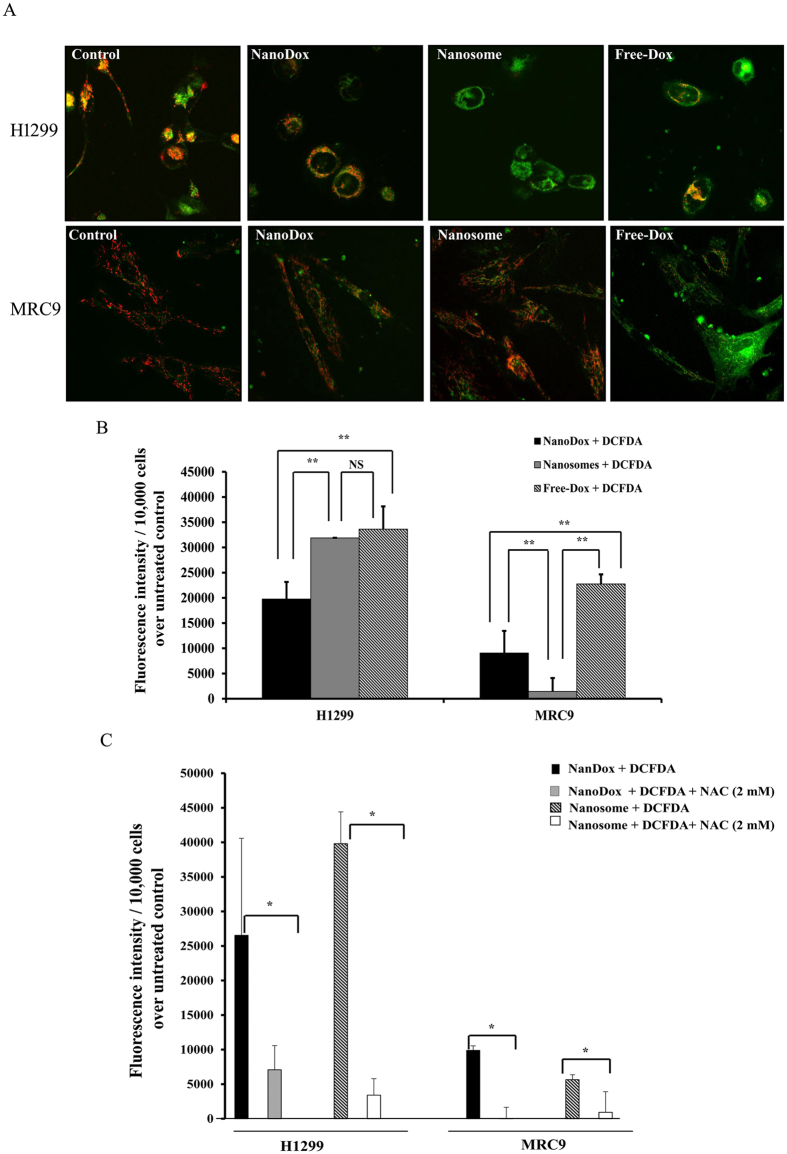
Nanosomes induce mitochondrial perturbation and ROS. (**A**) H1299 and MRC9 cells treated with NanoDox-, nanosome-, and freeDox were analyzed for change in mitochondrial potential using JC1 stain. Cells receiving no treatment served as controls. Nanosome treatment markedly perturbed the mitochondrial potential in H1299 cells compared to NanoDox and control cells. In MRC9 cells, the perturbance in the mitochondrial potential was less in nanosome-treated cells compared to NanoDox- and free-Dox-treated cells. (**B**) Analysis for intracellular ROS production showed nanosome-induced ROS level was significantly higher than in NanoDox and was comparable to free-Dox-induced ROS levels in H1299 cells. The nanosome-induced ROS levels however was significantly lower in MRC9 cells compared to NanoDox- and free-Dox-induced ROS levels. Untreated cells served as controls. Each bar represents the ROS status in each treatment normalized to untreated control + DCFDA (baseline) ± *SD*. (**C**) ROS production by nanosome and NanoDox was significantly abrogated by the antioxidant, NAC in both H1299 and MRC9 cells. * Represents *p* < 0.05, ** represents *p* < 0.0001, NS = not significant.

Studies have shown Dox-treatment induces mitochondrial damage and ROS accumulation in cancer cells^39^. Further, it is known that ROS can contribute to DNA damage^40^. Since, nanosome treatment resulted in DNA damage we measured the mitochondrial membrane potential using the cationic JC-1 dye (Fig. 7A) and ROS (Fig. 7B) assay in H1299 and MRC9 cells. In H1299 cells, disruption of mitochondrial potential was evident upon treatment with free-Dox, NanoDox, and nanosome compared to control (Fig. 7A). However, the highest disruption of mitochondrial potential was observed in the free-Dox and nanosome treatment group and was comparable between the two treatment groups. In MRC9 cells, the highest disruption of mitochondrial potential was observed in the free-Dox treatment group compared to all other treatment groups (Fig. 7A). Nanosome treatment showed relatively less disruption of mitochondrial potential compared to NanoDox treatment.

Analysis for ROS production revealed significant increase in H1299 cells treated with NanoDox, nanosome and free-Dox compared to MRC9 cells (Fig. 7B; *p* < 0.001). In H1299 cells, nanosome and free Dox-treatment showed the highest ROS levels compared to NanoDox (*p* < 0.001). ROS levels were comparable between nanosome and free-Dox-treatment with no significant difference observed. In MRC9 cells, free-Dox produced the highest level of ROS while nanosome produced the lowest ROS levels compared to NanoDox and free-Dox (Fig. 7B; *p* < 0.001) an observation that correlated with the mitochondrial disruption study results.

Next to confirm that the observed ROS production is specifically induced by nanosomes, we conducted ROS quenching studies in both H1299 and MRC9 cells. As shown in Fig. 7C, ROS induced by NanoDox and nansomes was significantly abrogated by the antioxidant, *N*-Acetylcysteine (NAC) in the two cell lines tested (*p* < 0.05). These results show that nanosomes perturb the mitochondrial membrane potential and induce ROS to a greater extent in cancer cells than in normal cells that culminate in increased DNA damage and apoptosis of cancer cells thus sparing normal cells.

### Nanosomes produce minimal cytotoxicity in human coronary artery muscle (HCASM) cells

**Figure 8:**
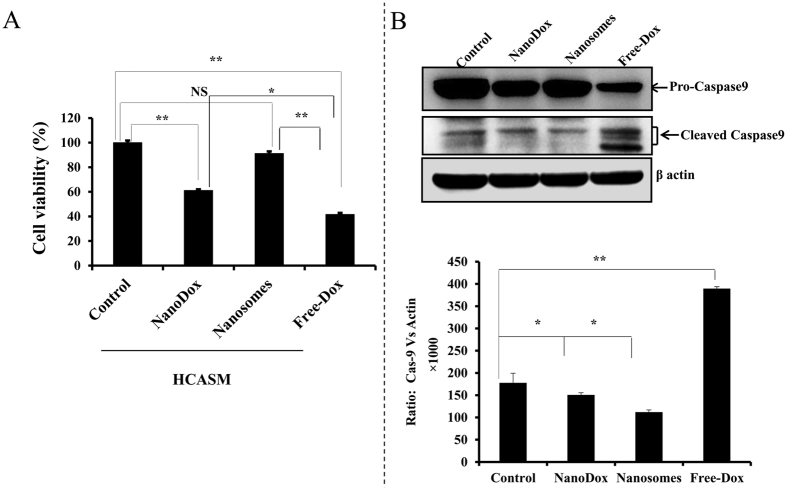
Nanosomes exhibit minimal cytotoxicity in human coronary aortic muscle (HCASM) cells. HCASM cells were treated with NanoDox, nanosomes, and free-Dox, each containing the equivalent of 5 μg Dox/well. Cell viability was determined at 24 h after treatment. Untreated cells served as controls (**A**). Treatment with NanoDox and free-Dox significantly reduced cell viability compare to controls. However, nanosome treatment exhibited minimal toxicity compared to controls. Each bar represents the percent viable cells after 24 h of treatment ± *SD* compared with untreated control. *Represents *p* < 0.05, **represents *p* < 0.0001, and NS = not significant. (**B**) Western blot shows caspase-9 activation was reduced in nanosome-treated cells compared to free-Dox and NanoDox-treated cells. β-actin was used as a loading control.

Cardiotoxicity is one of the serious side effects of doxorubicin that limits its use in cancer treatment^41^,^42^. Thus, we examined whether nanosomes and/or NanoDox would reduce the toxicity of Dox towards HCASM cells. Strikingly, the Dox-induced toxicity in HCASM cells was the least when Dox was delivered using nanosomes (91% cell viability) compared to control after 24 h of treatment. In contrast, Dox-induced toxicity was the highest in free- Dox (41% cell viability; *p* < 0.001) treatment followed by NanoDox (61% cell viability; *p* < 0.001) treatment compared to control (Fig. 8A). Correlating with the cell viability study results was the reduced activation of caspase-9 in nanosome- treated cells compared to NanoDox- and free-Dox-treated cells (Fig. 8B). The highest caspase-9 cleaved product was observed in free-Dox-treated cells compared to control (*p* < 0.001). These results clearly demonstrate that nanosomes are safe in protecting cardiac cells from Dox-induced toxicity compared to free-Dox, a feature that is of clinical importance in cancer treatment.

## Discussion

One among many challenges in effectively controlling cancer growth is the inefficient drug delivery and poor accumulation of the cytotoxic chemotherapy drug in the tumor. While several drug delivery systems have been developed and tested in preclinical studies, very few have advanced to clinical testing due to inherent limitations including toxicity to normal cells. Therefore, there is pressing need for testing new drug delivery system that are safe and exhibit preferential toxicity to tumor cells and spare normal cells.

Exosomes due to their size, structure, origin, and similarity with synthetic liposomes have recently garnered attention as delivery vehicle for anticancer drugs and molecules, such as siRNA^43^,^44^,^45^,^46^. Additionally, the presence of several adhesion proteins on the surface of exosomes and its tendency for enhanced fusion with recipient cells under low pH condition uniquely position exosomes to deliver therapeutics with increased efficiency and specificity to tumor cells^47^,^48^. Studies testing exosomes as natural drug delivery vehicle for cancer treatment have been reported^43^,^44^,^45^. In these studies, loading of drug or molecules onto exosomes has been accomplished through several methods, including sonication^43^, electroporation^43^,^44^, or by transfecting the cells with the molecules of interest and then isolating the exosomes loaded with those molecules from the transfected cells^21^,^44^. However, one major limitation of these studies is that there is no control over release of the drug. As a result there is a potential for having a burst release of the drug which while exhibiting tumor cell cytotoxicity will be limited in having incomplete efficacy. This in particular will be of significant concern when drugs such as doxorubicin that exhibits cardiac toxicity is administered. Furthermore, methods such as electroporation and sonication are likely to produce structural or physiological changes in the exosomes thus affecting their transfection efficiency^49^,^50^.

In the present study, we adopted a simplistic approach in which GNP-conjugated to Dox via a stimuli responsive linker (or NanoDox) was loaded onto exosomes by simple incubation to produce “nanosomes”. The surface charge of NanoDox is positive, while that of exosomes is negative. These opposing charges enhance the loading of NanoDox onto exosomes. In addition, the dynamic nature of the exosome’s lipoprotein membrane facilitates the import of NanoDox into its lumen. With our nansomes, we could demonstrate multiple molecules of NanoDox could be loaded into exosomes without causing any morphological changes in shape or size of the exosomes as shown by TEM imaging studies. Further, loading of NanoDox onto exosomes offered several advantages over directly loading free-Dox onto exosomes (Exo-Dox) in terms of the drug release kinetics and cell killing. Exo-Dox showed a rapid burst release of drug within the first 4 h of adding the complex to the tumor cells, and enhanced cell death (Supplementary Fig. S-6), a finding that concurred with previous report by Kim *et al*.^43^. In contrast, a delayed and sustained released of Dox up to 72 h producing cytotoxicity was observed when nanosomes was added to tumor cells. The delayed and sustainable cytotoxicity and better performance of nanosome indicates that, along with pH- controlled release, nanosomes take time to fuse with the recipient cells and release their content into the cytosol.

While we have been able to successfully load GNP-Dox onto exosomes, the mechanism by which GNP-Dox enter the exosomes is not known and is yet to be determined. Understanding the mechanisms of entry will enable methods to increase the loading efficiency. Further, in the present study we have not determined whether the distribution of the GNP-Dox in various compartments of the exosome such as in the bilayer membrane versus inside the core will affect the treatment outcome. These unanswered questions while of interest will be pursued in future studies that will advance the nanosome technology.

One of the major limitations of using doxorubicin in the clinic, despite its potency as an anticancer agent, is its non-specific toxicity to cardiomyocytes resulting in acute cardiotoxicity, even at very low doses. As a result administration of Dox is limited. Functional studies showed that nanosomes were not only potent in killing tumor cells that was comparable to free-Dox but also exhibited reduced toxicity toward both, normal fibroblast cells and HCASM cells compared with that of free-Dox and NanoDox. A similar observation was reported by Toffoli *et al*.^51^. The authors of that study using an *in vivo* tumor model showed exosome-mediated Dox delivery had superior biodistribution and efficacy along with reduced normal tissue toxicity compared to free-Dox. Our study results thus provide a method to circumvent cardiotoxicity while enabling tumor killing. While the study results are promising it is to be approached with caution as the cardiotoxicity of nanosomes when administered *in vivo* remains unknown. Studies in our laboratory are currently in progress for testing the efficacy of the nanosomes *in vivo* in a lung cancer xenograft model.

Studies investigating the underlying molecular mechanism by which nanosomes exerted its cytotoxicity revealed induction of G2 phase cell cycle arrest, activation of the apoptosis cascade, and DNA damage. The DNA damage was contributed by mitochondrial membrane perturbation and ROS production that was significantly abrogated in the presence of the antioxidant, NAC. The increased ROS production in nanosome-treated cells compared to NanoDox is likely due to prolonged release of the drug over time thereby producing a sustained impact on the mitochondria resulting in heightened ROS. In contrast, drug release in NanoDox is relatively short and quick resulting in lower mitochondria disruption and ROS production.

Finally, while cancer patient-derived, exosome-based drug delivery system is being considered as an attractive tool for the development of personalized medicine, our studies showed the potential hazard in using tumor-derived exosomes as drug delivery vehicle for cancer treatment. We observed H1299-derived exosomes but not MRC9-derived exosomes when added to naïve H1299 cells enhanced cell proliferation. Similar observations were reported by Saari *et al*.^52^ in their study testing the efficacy of exosome-paclitaxel complexes on prostate cancer cell lines. Thus, while use of exosomes as drug delivery vehicle is attractive it is imperative to use caution in selecting the exosome source and assess the benefit to risk prior to its application especially when planning for cancer treatment.

Whether the nanosome-based drug delivery approach is limited to Dox or could be used for other chemotherapeutics is of interest. Preliminary studies in our laboratory testing cisplatin (CDDP) conjugated to GNP could also be efficiently loaded into exosomes suggesting that our nanosome technology has broad applicability in loading different anticancer drugs that are hydrophilic or hydrophobic in nature (data not shown). Although not explored in the present study, multiple drugs and/or targeting molecules can be added to the GNPs prior to loading onto exosomes. This will allow the use of nanosomes for both imaging and therapy. While we have convincingly demonstrated nanosomes are safe and efficacious *in vitro*, it will be important to investigate the efficacy and safety of the nanosomes *in vivo* in the future. Another important factor to consider is whether tumor-targeting will be important for the nanosomes to exhibit efficacy *in vivo* The *in vivo* studies while of interest are beyond the scope of present study.

The present study was an attempt to take advantage of the cargo-carrying capacity of exosomes across different cells, often at large distances, to deliver drug/genes to the cancer cell. The results of the present study provide proof of the concept that exosomes can be used as ideal carriers for drug delivery system. Although, the science of exosomes is still in its infancy, and there are certain challenges such as ability to lyophilize and reconstitute in water, making them amenable for prolonged storage and use must be addressed and more studies are needed before this approach is used in clinical practice.

## Additional Information

**How to cite this article**: Srivastava, A. *et al*. Nanosomes carrying doxorubicin exhibit potent anticancer activity against human lung cancer cells. *Sci. Rep.* **6**, 38541; doi: 10.1038/srep38541 (2016).

**Publisher's note:** Springer Nature remains neutral with regard to jurisdictional claims in published maps and institutional affiliations.

## Supplementary Material

Supplementary Information

## Figures and Tables

**Table 1:** Determination of elemental Gold (Au) present in NanoDox and nanosomes and intake of NanoDox and nanosomes by H1299 cells in 24 h was measured by using ICP-MS.

Sample	Gold (Au) content in ppb
NanoDox	98
Nanosomes	236
Cell + NanoDox	14.55
Cells + Nanosomes	89.02
