# Supplementary material for: Nanosomes carrying doxorubicin exhibit potent anticancer activity against human lung cancer cells
Source: Sci Rep. 2016 Dec 12;6:38541. doi: 10.1038/srep38541 (PMC5150529; doi:10.1038/srep38541)
Supplement: Supplementary Information [file srep38541-s1.pdf]

Nanosomes carrying doxorubicin exhibit potent anticancer activity against human lung cancer cells

Akhil Srivastava [a,h,+], Narsireddy Amreddy[a,h,+], Anish Babu[a,h], Janani Panneerselvam [a,h], Meghna Mehta [b,h], Ranganayaki Muralidharan [a,h], Allshine Chen[c], Yan Daniel Zhao[c,h], Mohammad Razaq[d,h], Natascha Riedinger[e], Hogyoung Kim[f], Shaorong Liu[g,h], Si Wu[g,h], Asim B. Abdel-Mageed[f], Anupama Munshi[b,h], Rajagopal Ramesh[a,h,i*]

Departments of [a]Pathology, [b]Radiation Oncology, [c]Epidemiology and Statistics, [d]Medicine, University of Oklahoma Health Sciences Center, Oklahoma City, OK, USA; [e]Boone Pickens School of Geology, Oklahoma State University, Stillwater, OK, USA; [f]Department of Urology, Tulane University School of Medicine, New Orleans, LA, USA; [g]Department of Chemistry and Biochemistry, University of Oklahoma, Norman, OK; [h]Stephenson Cancer Center, University of Oklahoma Health Sciences Center, Oklahoma City, OK, USA; [i]Graduate Program in Biomedical Sciences, University of Oklahoma Health Sciences Center, Oklahoma City, OK, USA.

[+] The first two authors contributed equally to the work.

***Address of Correspondence:** Rajagopal Ramesh, Department of Pathology, Stanton L. Young Biomedical Research Center, Suite 1403, 975 N.E., 10th Street, Oklahoma City, OK 73104, USA; Phone: (405) 271-6101; Email: rajagopal-ramesh@ouhsc.edu

Supplementary Section:

1. Isolation of exosomes: Exosomes were isolated using a modified differential high speed and ultracentrifugation method of as described in supplementary section Briefly, the medium collected from the cultured cells were centrifuged at 3000 × g for 10 min (Beckman Coulter Avanti J-26S, Beckman Coulter Life Sciences, Indianapolis, IN) to remove dead cells. The supernatant was then centrifuged at 10,000 × g for 20 min to remove all cellular debris. The supernatant was filtered through a 0.22 µm Millex® Syringe filter (EMD Millipore, Cork, Ireland). The filtrate was centrifuged at a speed of 100,000 ×g for 90 min in a Beckman Optima™ XPN ultracentrifuge (Beckman Coulter). Finally the exosome pellet was washed once with PBS (pH 7.4) and stored at -80 ºC until use. All centrifugation steps were performed at 4 ºC.

2. Characterization of exosomes:

Exosomes concentration and size. The concentration and size of exosomes was measured in the isolates from the cell culture medium by using the qNano system (Izon Science Ltd., Cambridge, MA). Aliquots containing isolated vesicles were passed through nanopore by using Tunable Resistive Pulse Sensing (TRPS) technology [1]. Calibration particles of 50-200 nm were used to optimize the accuracy of detection before testing the actual samples. For analysis, diluted sample (40 µl) was injected to measure the concentration and size of exosomes. The sizes and concentrations of particles were calculated using the software provided by Izon (version 3.2.2.268).

Transmission electron microscope (TEM) imaging. Purified exosomes were suspended in PBS, and were fixed in 1% paraformaldehyde for 30 min at room temperature. The fixed exosomes were dropped onto copper electron microscope (EM) grids and were allowed to dry for 60 min. Then, the exosomes were stained with 1 % phosphotungstic acid (PTA) and were visualized by TEM at an acceleration voltage of 80 kV (Hitachi 7600,Tokyo, Japan)

Detection of exosome-related protein markers. To analyze the purity of exosome preparations, western blotting (described later) was performed with 100 µg of exosomes isolated from H1299 and MRC9 cells and primary antibodies against exosome markers CD63 (1:1000; System Biosciences), CD81 (1:1000; System Biosciences), AGO2 (1:1000; Abcam, Cambridge, MA), Hsp90B1 (1:1000; Abcam) and TSG101 (1:1000; Santa Cruz Biotechnology, Dallas, TX).

3. Western blotting:

Primary antibodies against caspase-9 and γH2AX (1:1000; Cell Signaling Technology Inc.; Beverly, MA) and beta actin (1:2000; Sigma Chemicals) were purchased and used as recommended by the manufacturers. Proteins were detected using the appropriate secondary antibodies (Santa Cruz Biotechnology, Inc., and Jackson Immuno-Research Laboratories, Inc., West Grove, PA) and an enhanced chemiluminescence kit (Thermo Scientific). Protein levels were detected using a chemiluminescence imaging system (Syngene, Frederick, MD) and quantified using Image Quant software.

4. Comet assay:

Briefly, 1×10^5 H1299 cells were seeded and treated with free-Dox, NanoDox and nanosomes each containing the equivalent of 5 µg of Dox. After 24 h of treatment the cells were collected and processed per manufacturer recommended protocol. Images of comets were taken with an epifluorescence microscope (Nikon) and were analyzed using Casplab comet assay software (CASP v1.2.3b2; www.Casplab.com). A total of thirty cells per treatment group were analyzed for determination of the olive tail moment as an indicator of DNA damage

References:

1. Coumans, F. A. W. *et al*. Reproducible extracellular vesicle size and concentration determination with tunable resistive pulse sensing, *J. Extracell. Vesicles* **10**, 25922 (2014)

Supplementary Figures

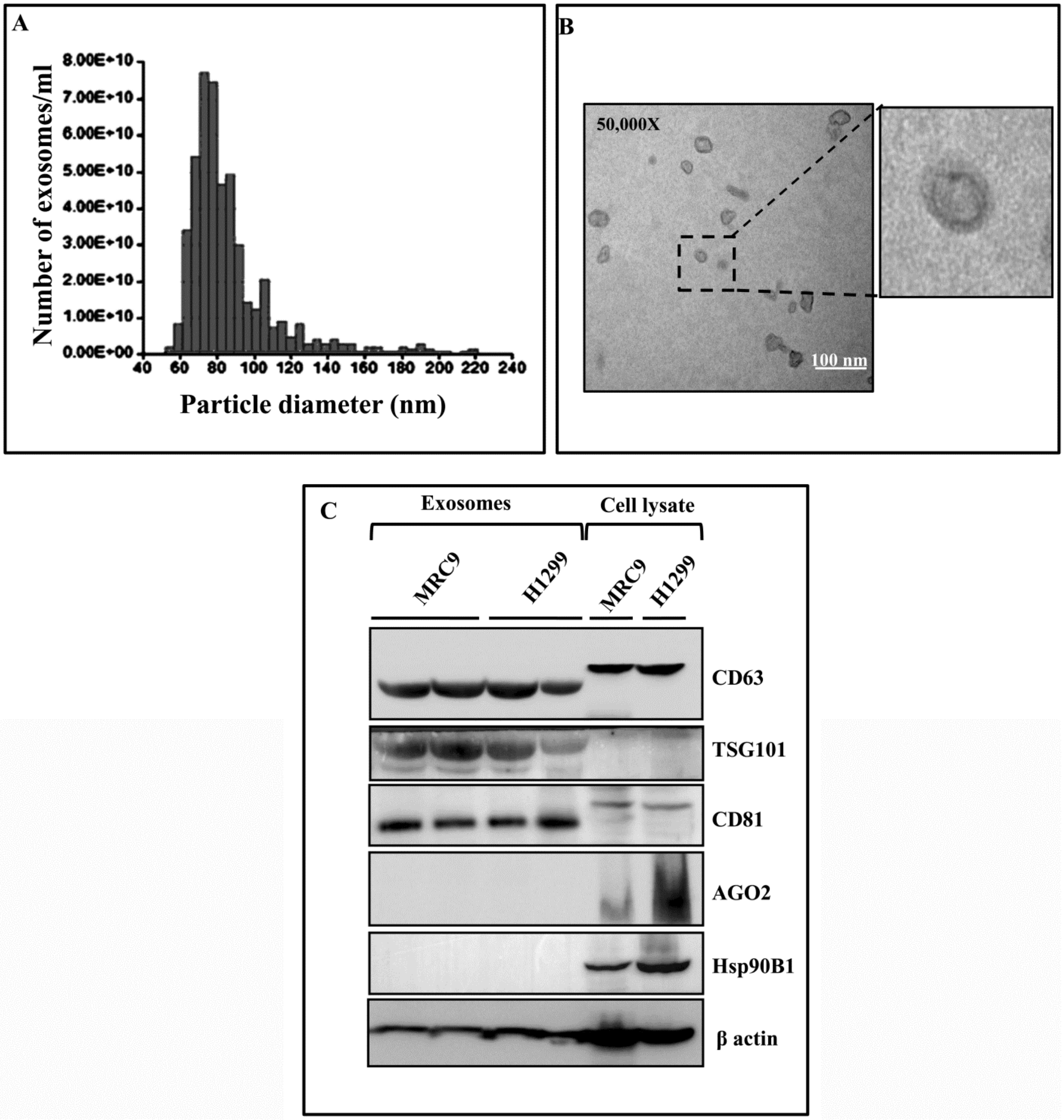

Figure S-1. Characterization of isolated exosomes. ***A***, Particle size distribution of exosomes measured with the IZON qNano particle counter. ***B***, TEM images of isolated exosomes, showing size and shape. Scale bar, 100 nm. ***C***, Western blots with exosome-associated markers in cell lysate and exosomes isolated from MRC9 and H1299 cell lines.

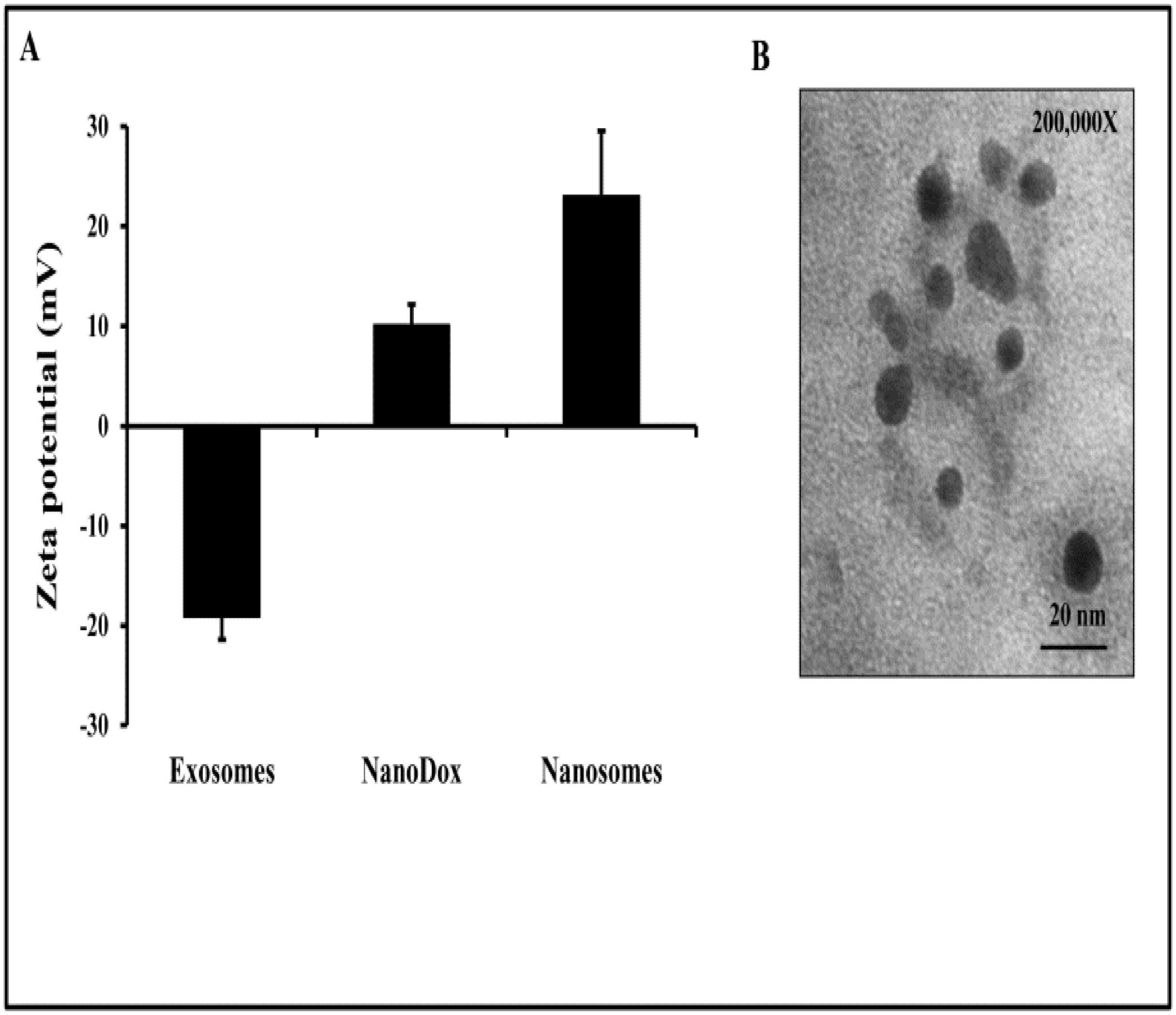

Figure S-2.Characterization of nanosomes prepared with exosomes isolated from MRC9 cells. ***A***, zeta potential analysis shows changes in surface charge in exosomes, NanoDox, and nanosomes. ***B***, TEM image of nanosome showing NanoDox present in the bilayer and lumen of the exosome; magnification 200,000X; Scale bar, 20 nm;

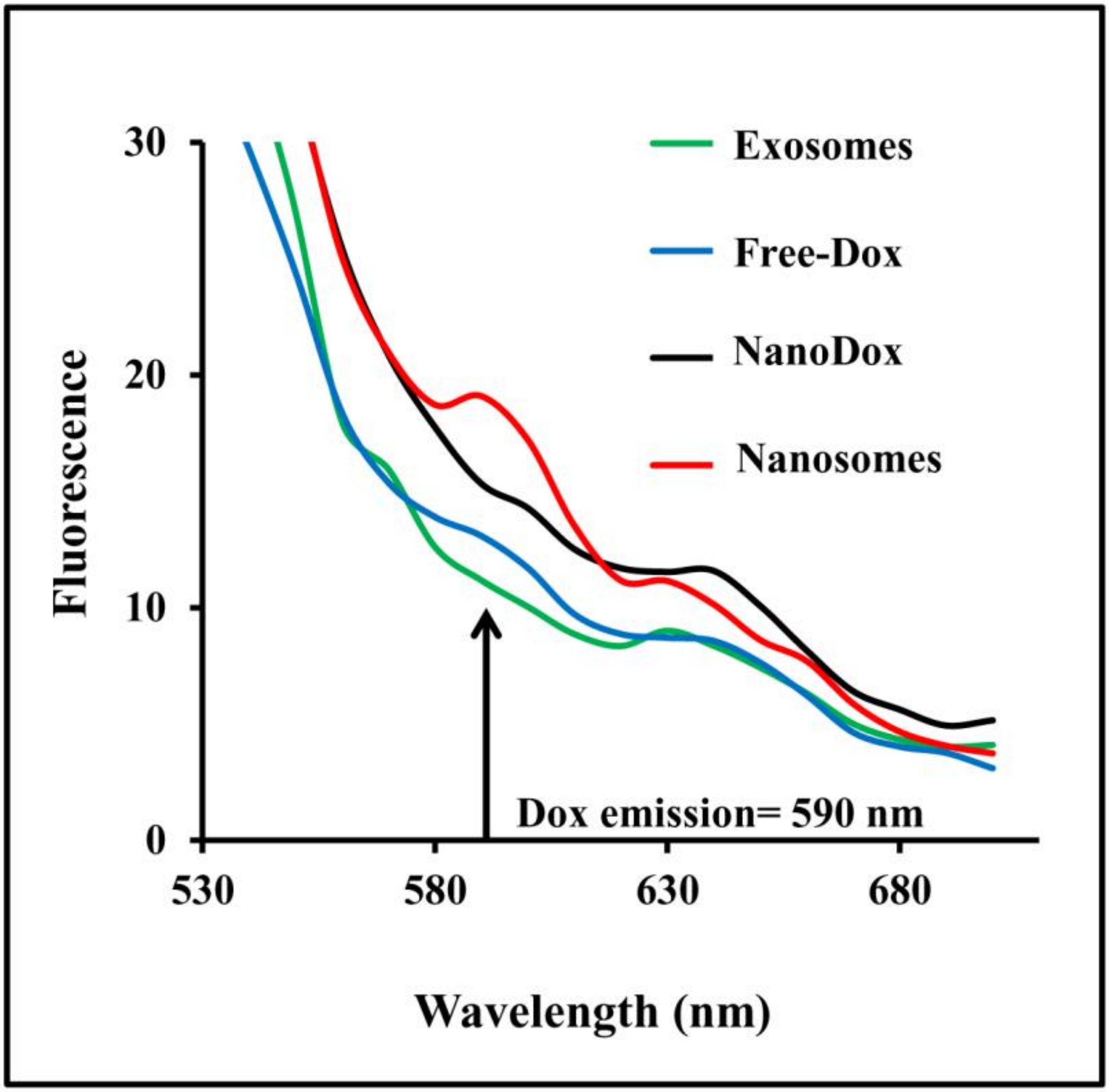

Figure S-3. Measurement of Doxorubicin (Dox) by fluorescence spectral analysis. Presence of Dox as evidenced by the shift in the peak at around 590 nm was detected in the nanosomes- and NanoDox-treated H1299 cells. Free -Dox was used as positive control and drug-free exosomes as negative control.

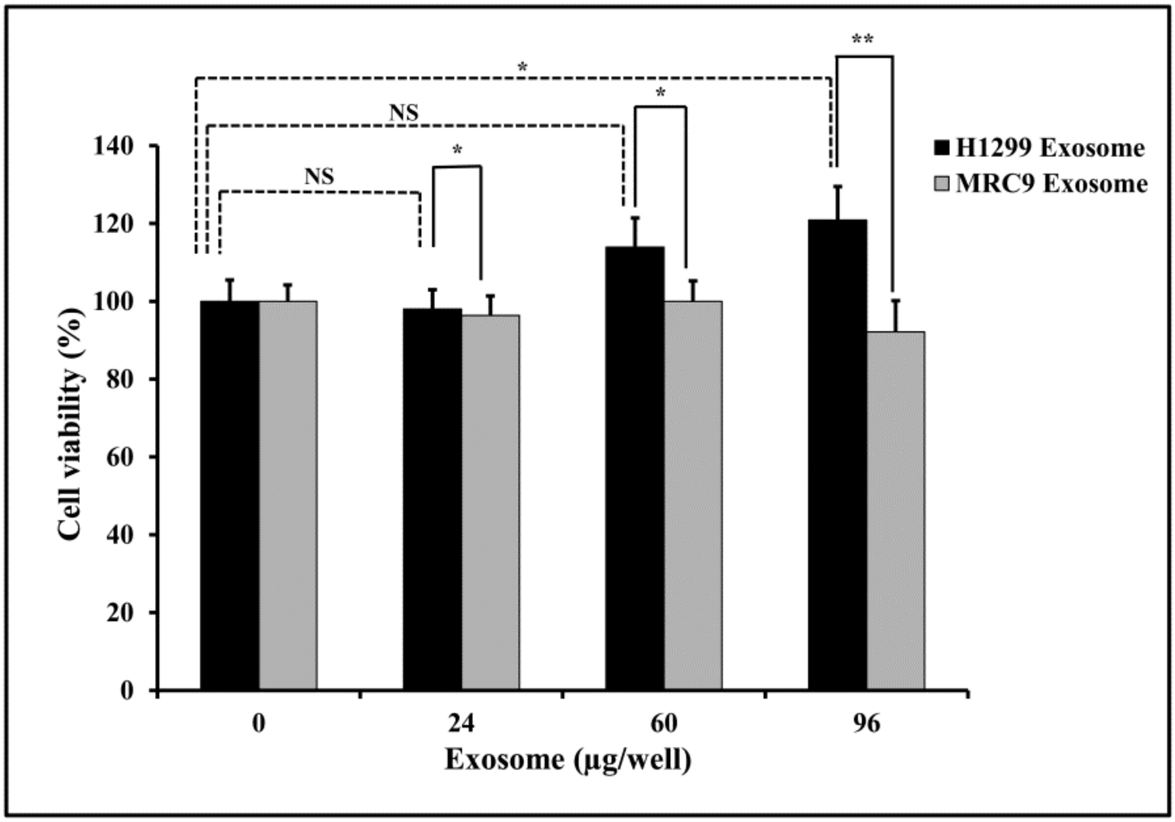

Figure S-4. Cell viability assay with H1299 cells treated with pure exosomes isolated from H1299 and MRC9 cells. Addition of exosomes derived from H1299 cells but not from MRC9 cells increased cell viability. Each bar represents the percent viable cells after 24 h of treatment ± *SD*. * represents $p<0.05$, ** represents $p<0.0001$, NS = not significant.

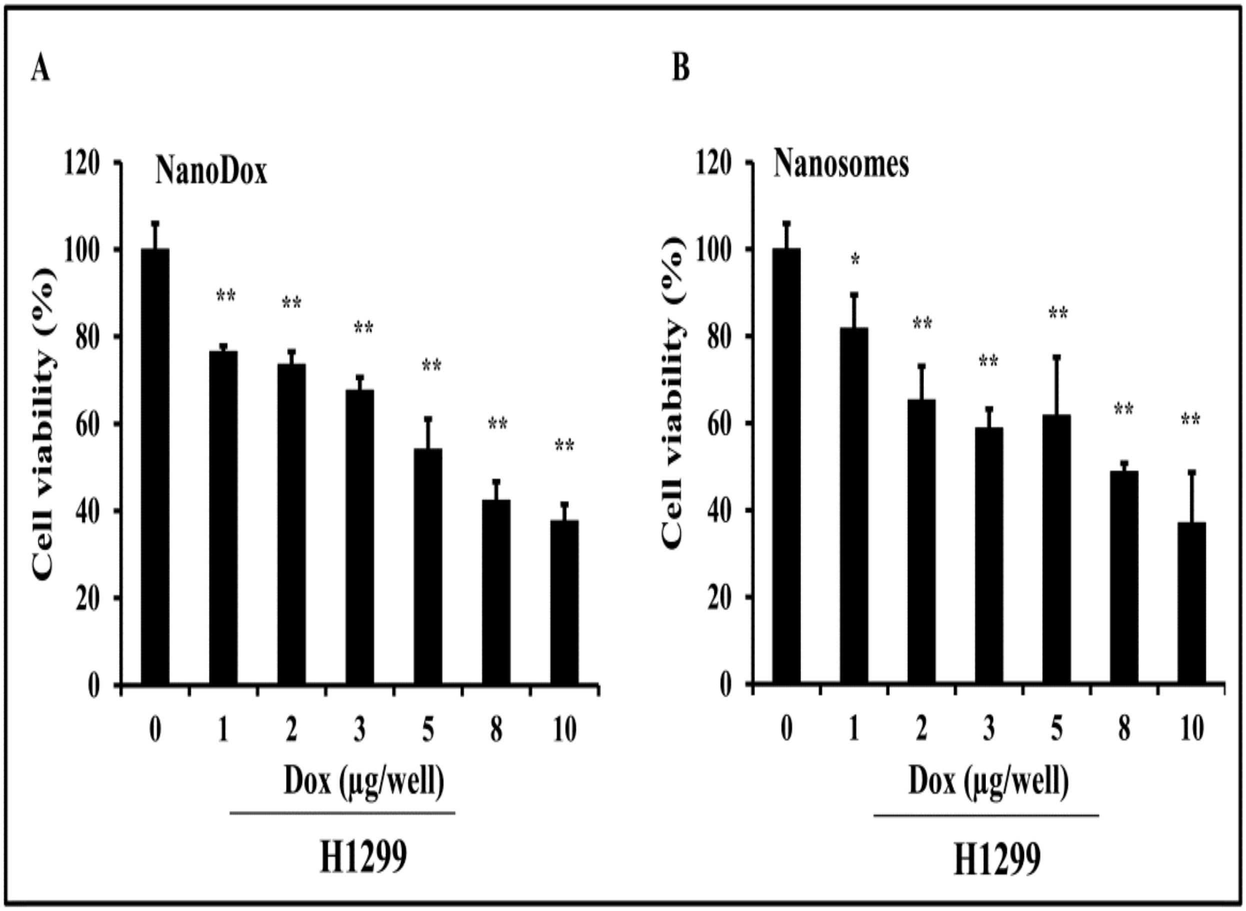

Figure S-5. Cell viability assay with different doxorubicin (Dox) concentrations present in ***A***, NanoDox and ***B***, nanosomes on H1299 cells. A dose-dependent reduction in cell viability was observed in both NanoDox and nanosome treatments. Each bar represents the percent viable cells after 24 h of treatment ± *SD*. * represents $p<0.05$, ** represents $p<0.0001$.

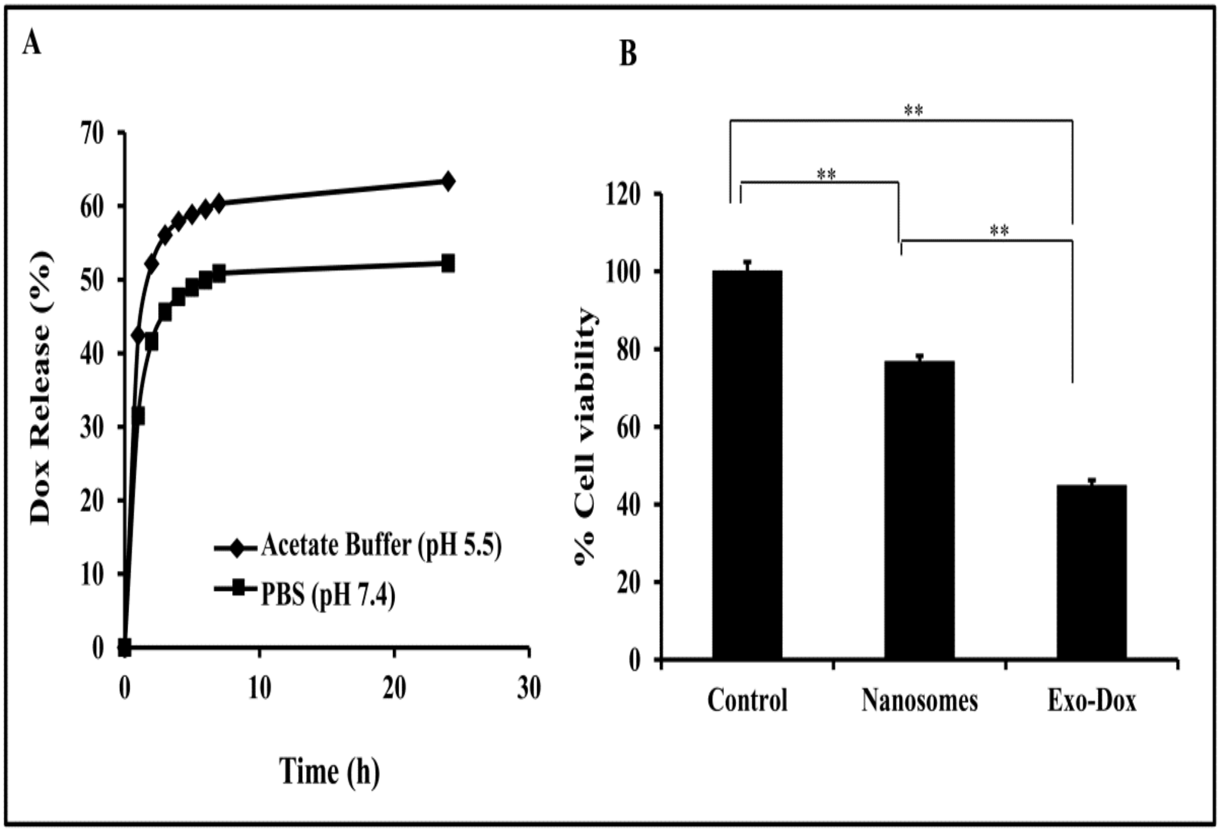

Figure S-6.*A*, *In vitro* drug release kinetics from free-Dox-loaded exosomes (Exo-Dox) in PBS buffer (pH 7.4) and acetate buffer (ABS; pH 5.5). A rapid burst release of Dox from Exo-Dox is observed within 4 h when incubated in the two buffers and was comparable. ***B***, Cell viability showed Exo-Dox produced greater reduction in H1299 cell viability compared to nanosomes. Each bar represents the percent viable cells after 24 h of treatment ± *SD* compared with untreated control. ** represents $p<0.0001$.
